# Relationship of Ocular Microcirculation, Measured by Laser Speckle Flowgraphy, and Silent Brain Infarction in Primary Aldosteronism

**DOI:** 10.1371/journal.pone.0117452

**Published:** 2015-02-12

**Authors:** Hiroshi Kunikata, Naoko Aizawa, Masataka Kudo, Shunji Mugikura, Fumihiko Nitta, Ryo Morimoto, Yoshitsugu Iwakura, Yoshikiyo Ono, Fumitoshi Satoh, Hidetoshi Takahashi, Sadayoshi Ito, Shoki Takahashi, Toru Nakazawa

**Affiliations:** 1 Department of Ophthalmology, Tohoku University Graduate School of Medicine, Sendai, Japan; 2 Department of Retinal Disease Control, Tohoku University Graduate School of Medicine, Sendai, Japan; 3 Division of Nephrology, Endocrinology, and Vascular Medicine, Department of Medicine, Tohoku University Graduate School of Medicine, Sendai, Japan; 4 Department of Diagnostic Radiology, Tohoku University Graduate School of Medicine, Sendai, Japan; 5 Department of Advanced Ophthalmic Medicine, Tohoku University Graduate School of Medicine, Sendai, Japan; Tokyo Institute of Technology, JAPAN

## Abstract

**Purpose:**

Recent studies have shown that the risk of cerebro- and cardiovascular events (CVEs) is higher in patients with primary aldosteronism (PA) than in those with essential hypertension (EH), and that silent brain infarction (SBI) is a risk factor and predictor of CVEs. Here, we evaluated the relationship between findings from laser speckle flowgraphy (LSFG), a recently introduced non-invasive means of measuring mean blur rate (MBR), an important biomarker of ocular blood flow, and the occurrence of SBI in patients with PA.

**Methods:**

87 PA patients without symptomatic cerebral events (mean 55.1 ± 11.2 years old, 48 male and 39 female) were enrolled in this study. We measured MBR in the optic nerve head (ONH) with LSFG and checked the occurrence of SBI with magnetic resonance imaging. We examined three MBR waveform variables: skew, blowout score (BOS) and blowout time (BOT). We also recorded clinical findings, including age, blood pressure, and plasma aldosterone concentration.

**Results:**

PA patients with SBI (15 of 87 patients; 17%) were significantly older and had significantly lower BOT in the capillary area of the ONH than the patients without SBI (*P* = 0.02 and *P* = 0.03, respectively). Multiple logistic regression analysis revealed that age and BOT were independent factors for the presence of SBI in PA patients (OR, 1.15, 95% CI 1.01–1.38; *P* = .03 and OR, 0.73, 95% CI 0.45–0.99; *P* = .04, respectively).

**Conclusion:**

PA patients with SBI were older and had lower MBR BOT than those without SBI. Our analysis showed that age was a risk factor for SBI, and that BOT was a protective factor, in patients with PA. This suggests that BOT, a non-invasive and objective biomarker, may be a useful predictor of SBI and form part of future PA evaluations and clinical decision-making.

## Introduction

Primary aldosteronism (PA), also known as primary hyperaldosteronism, is characterized by bilateral adrenal hyperplasia or aldosterone-producing adenomas causing overproduction of the mineralocorticoid hormone aldosterone, without excessive renin secretion.[1,2] PA patients are becoming more prevalent and now account for up to 10% of cases of hypertension in selected populations, constituting the most frequent endocrinal cause of hypertension and a major problem worldwide.[3,4] Aldosterone causes an increase in sodium and potassium excretion in the renal tubules, leading to water retention and a subsequent increase in blood pressure.

There are many reports showing that prolonged exposure to high aldosterone concentrations negatively affects cardiovascular tissues independently of blood pressure (BP).[5,6,7,8] Namely, a higher risk of *cerebro- and cardiovascular* events (CVEs) has been observed in PA patients than in those with essential hypertension (EH).[5,9,10] Silent brain infarctions (SBIs), despite not causing identifiable symptoms and often remaining unnoticed by patients, still cause damage to the brain and place the patient at increased risk for both transient ischemic attacks and major strokes.[11] However, there are currently no reports on the clinical relationship between PA and SBI.

Recently, laser speckle flowgraphy (LSFG) has been introduced as a non-invasive and quick means of measuring mean blur rate (MBR) in the human retina, an important quantitative biomarker of ocular blood flow. Monitoring MBR over time with LSFG allows us to examine changes in the optic microcirculation of a single eye, and to compare blood flow waveform parameters between eyes.[12,13] Blowout time (BOT), one of the variables of this waveform analysis, has previously been reported to be useful in evaluating early atherosclerotic changes or aging of the microcirculation.[14,15,16] Many previous studies have also shown a close relationship between CVEs and qualitative measurements of retinal microvascular abnormalities.[17,18,19,20] We hypothesized that the ocular circulatory waveform could reveal SBIs because atherosclerosis causes microcirculation damage in both the eye and brain [17,21,22]. Therefore, clinical measurement values in the eye, such as the ocular MBR waveform, should reflect the status of the brain. We thus evaluated the relationship between LSFG findings and the occurrence of SBI in patients with PA.

## Materials and Methods

### Setting and Design

This was an institutional, case series. Subjects were recruited from patients referred to the Department of Ophthalmology of Tohoku University Hospital, where examinations and follow-up were performed.

### Patients

The study comprised 87 eyes of 87 patients (48 men and 39 women, mean age ± standard deviation: 55.1 ± 11.2 years) with PA who were treated at Tohoku University Hospital between April 2012 and August 2013. PA was diagnosed according to previously reported criteria.[23] The study protocol was approved by the ethics committee of Tohoku University School of Medicine. All participants provided their written informed consent to participate in this study. The research was conducted according to the provisions of the Declaration of Helsinki, 1995 (as revised in Edinburgh, 2000).

### Detection of Silent Brain Infarction

Magnetic resonance imaging (MRI), performed with one of two 1.5 Tesla MRI units, was used to detect SBI.[24] SBI was defined as the presence of lesions ≥ 3 mm in diameter in their widest dimension, cerebrospinal fluid signal characteristics in T1- and T2-weighted MRI images, and a hyperintense rim surrounding the lesion in fluid attenuated inversion recovery images.[24,25] Lesions of diameter > 15 mm were excluded from the SBI analysis. Among detected lesions ≥ 3 mm, a particular effort was made to differentiate cavitated lacunes from large dilated perivascular spaces based on their location. Lesions located in the lower third of the basal ganglia were not considered to be infarcts. We regarded patients as positive for SBI when ≥ 1 area was visible on the MRI image. Judgements were made independently by two neuroradiologists (S.M. and S.T.) who were blinded from the clinical and laboratory data of the subjects. Consensus was used to solve disagreements between judges. The subjects were divided into 2 groups: those with SBI lesions (the SBI group) and those without any lesions (the non-SBI group).

### Measurement of Physical and Hematological Findings

Systolic blood pressure and diastolic blood pressure (SBP and DBP) were measured after the patients had rested in a sitting position for 10 min. Measurements were made in the left brachial artery at the height of the heart by an automated blood pressure monitor (HEM-759E, Omron Corporation, Kyoto, Japan), following which measurements of ONH circulation were made with LSFG. At the first visit, tests for plasma aldosterone concentration (PAC) (ng/dL), plasma renin activity (PRA) (ng/mL/h), serum creatinine, serum sodium: Na (mEq/L), serum potassium: K (mEq/L), serum chlorine: Cl (mEq/L), estimated glomerular filtration rate (eGFR) (ml/min/1.73m^2^), total cholesterol (T-chol) (mg/dl), high-density lipoprotein cholesterol (HDL-C) (mg/dl), urinary sodium, urinary potassium, urinary albumin excretion (UAE), and urinary aldosterone (U-aldosterone) (μg/day) were performed in the morning after a 10-min rest in a sitting position. PAC and PRA were measured by radioimmunoassay; the former was measured with a SPAC-S Aldosterone Kit (TFB Inc., Tokyo, Japan), and the latter with a Renin Riabead Kit for PRA (Dainabot Co. Ltd., Tokyo). A diagnosis of PA was confirmed when the aldosterone-to-renin ratio exceeded 20 after the administration of 50-mg dose of captopril. Upon diagnosis of PA, antihypertensive agents were limited to a calcium channel blocker and/or an α1-blocker during the PA workup.[23,26] None of the patients had concurrent cortisol-producing adenoma, confirmed by an overnight 1-mg dexamethasone suppression test (DST 1 mg). Computed tomography (CT) scans were performed with a 64 channel multi-detector row CT (MDCT; Somatom Cardiac Sensation, Siemens, Germany) that analyzed the adrenal glands in contiguous 1.0 mm-thick slices at 0.5-mm intervals. All patients were treated according to the results of adrenal venous sampling (AVS) as previously reported.[26,27] Patients with unilateral disease diagnosed by AVS underwent laparoscopic adrenalectomy, while those with bilateral hyperaldosteronism were treated with a pharmacological regimen that included mineralocorticoid receptor antagonists (spironolactone or eplerenone).

### Measurement Using Laser Speckle Flowgraphy

Before LSFG measurement, the pupils of each subject were dilated with 0.5% tropicamide and 0.5% phenylephrine hydrochloride. The details of the underlying principles of LSFG (Softcare, Fukutsu, Japan) have been described in previous reports.[28,29] Briefly, the LSFG device consists of a fundus camera equipped with a diode laser (wavelength 830 nm) and an ordinary charge-coupled device camera (750 × 360 pixels). MBR, the relative velocity of blood flow, is derived from the pattern of speckle contrast produced by the interference of a laser scattered by blood cells moving in the ocular fundus.[30] MBR images are acquired continuously at the rate of 30 frames per seconds in a 4-second time period and transferred to a computer file. Equipped analysis software synchronizes the captured MBR images in each cardiac cycle, and averages the MBR in each heartbeat to produce a heartbeat map of the ONH. The LSFG software then automatically divides this map into the large vessel and capillary areas. Finally, the MBR waveform of a heartbeat in capillary area of the ONH is determined and three waveform analysis parameters were calculated: skew, blowout score (BOS), and BOT.[12,14,15,16] Three measurements of ONH circulation were made with LSFG and averaged for use in the statistical analysis. In this study, only waveform changes in the capillary area of the ONH were examined.

### Statistical Analysis

A Mann-Whitney U test and a chi-square test were used to determine the significance of differences between the groups. Spearman’s rank correlation test was used to evaluate single correlations between variables (age and tissue BOT). Logistic regression analysis was performed to determine the independent variables contributing to the occurrence of SBI in PA patients. A receiver operating characteristic (ROC) curve analysis was performed to assess the ability of age and tissue BOT to predict the occurrence of SBI (the area under the ROC curve; AUC). The statistical analyses were performed with JMP software (Pro version 10.0.2, SAS Institute Japan Inc., Tokyo, Japan). The significance level was set at *p* < 0.05.

## Results

**Fig 1:**
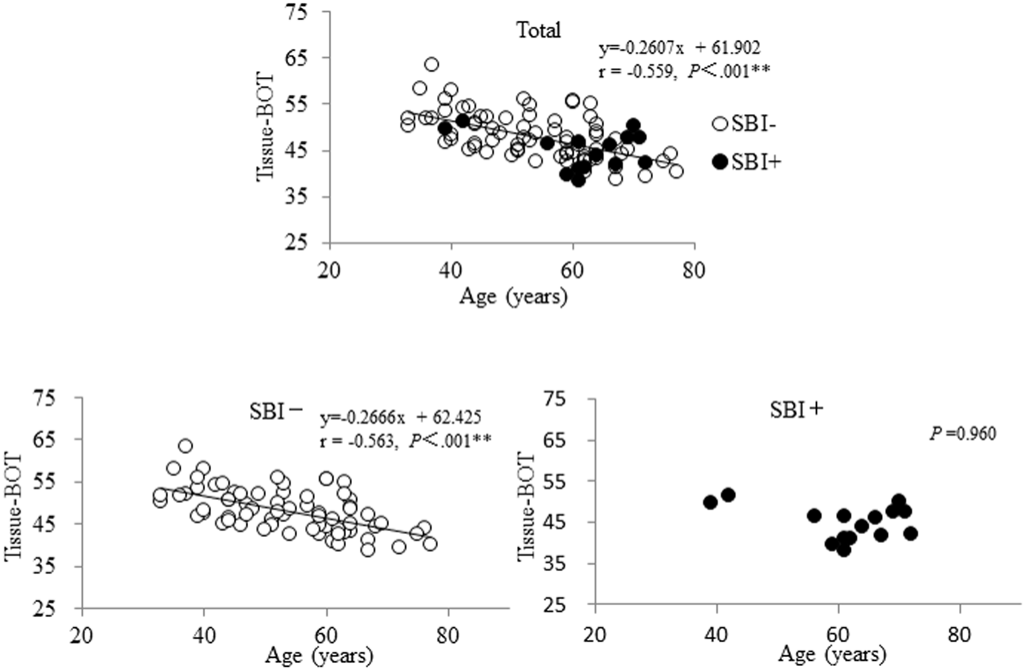
Relationship between age and blowout time in patients with primary aldosteronism. All patients with primary aldosteronism (PA) showed a correlation between tissue blowout time (BOT) and age (r = -0.559, *P*＜.001, upper). In PA patients without SBI, tissue BOT was closely correlated with age (r = -0.563, *P*＜.001, left), while in PA patients with SBI, tissue BOT was not correlated with age (*P* =. 96, right).

The systemic and ocular parameters obtained in this study are shown in Table 1 (mean ± standard deviation). There were 15 patients with SBI out of a total of 87 PA patients (17%). There were significant differences between the SBI and non-SBI groups in age, HDL-C and LSFG findings for tissue BOT (*P* = 0.02, *P* = 0.01 and *P* = 0.03, respectively); namely, age and HDL-C were higher and tissue BOT was lower in the SBI group. HDL-C was not correlated with age or tissue BOT in the entire group of PA patients (r = -0.12, *P* = 0.29 and r = -0.03, *P* = 0.78, respectively), but there was a correlation between tissue BOT and age (r = -0.559, *P*＜.001, Fig. 1). In PA patients without SBI, tissue BOT was well correlated with age (r = -0.563, *P*＜.001), while in PA patients with SBI, tissue BOT was not correlated with age (*P* =. 96).

**Table 1:** Characteristics of eyes with primary aldosteronism with and without silent brain infarction.

	SBI-	SBI+	*P* value
Number of eyes	72	15	-
Age	53.7 ± 11.1	61.4 ± 9.6	0.015*
Sex (M: F)	37: 35	11: 4	0.110
Physical findings			
BMI (kg/m^2^)	26.1 ± 3.6	25.4 ± 3.2	0.492
SBP (mmHg)	149.7 ± 22.6	155.5 ± 22.3	0.429
DBP (mmHg)	92.3 ± 16.0	85.6 ± 16.9	0.210
Hematological findings			
Na (mEq/L)	141.8 ± 1.8	142.2 ± 2.1	0.415
K (mEq/L)	3.8 ± 0.5	3.5 ± 0.8	0.081
Cl (mEq/L)	103. 8 ± 2.4	103.1 ± 3.1	0.279
PRA (ng/ml/hr)	0.88 ± 1.5	0.28 ± 0.4	0.165
PAC (ng/dl)	22.1 ± 11.4	28.1 ± 13.5	0.096
eGFR (ml/min/1.73m^2^)	75.2 ± 15.2	69.7 ± 26.4	0.301
T-chol (mg/dl)	193.8 ± 31.9	180.1 ± 40.9	0.199
HDL-C (mg/dl)	54.2 ± 15.1	42.6 ± 13.5	0.012*
U-aldosterone (μg/day)	17.7 ± 11.4	15.9 ± 9.9	0.564
LSFG parameters			
Tissue BOT	48.1 ± 5.1	44.9 ± 4.1	0.026*
Tissue BOS	74.5 ± 8.0	70.3 ± 6.7	0.061
Tissue Skew	13.0 ± 2.3	14.1 ± 2.8	0.121

BMI = body mass index, BOS = blowout score, BOT = blowout time, DBP = diastolic blood pressure, eGFR = estimated glomerular filtration rate, HDL-C = high-density lipoprotein cholesterol, PAC = plasma aldosterone concentration,

PRA = plasma renin activity, SBP = systolic blood pressure, T-chol = total cholesterol, U = urinary, LSFG = laser speckle flowgraphy

Differences between groups were analyzed with the Mann-Whitney U test. The chi-square test was used for frequency data on sex.

A multiple logistic regression analysis including age, SBP, eGFR, PRA, PAC, K, HDL-C and U-aldosterone as explanatory variables revealed that age was an independent risk factor against the presence of SBI in PA patients (OR, 1.15, 95% CI 1.01–1.38; *P* =. 03) (Table 2). The AUC for age for SBI was 0.71 (95% confidence interval (CI) 0.54–0.83; *P* =. 01) and the optimal cutoff value for age was determined to be 61 years. A multiple logistic regression analysis including tissue BOT, SBP, eGFR, PRA, PAC, K, HDL-C and U-aldosterone as explanatory variables revealed that tissue BOT was an independent protective factor against the presence of SBI in PA patients (OR, 0.73, 95% CI 0.45–0.99; *P* =. 04) (Table 3). The AUC for tissue BOT for SBI was 0.68 (95% CI 0.52–0.80; *P* =. 02) and the optimal cutoff value for tissue BOT was determined to be 42.2. There was no statistical difference between age and tissue BOT in predictive ability for SBI (*P* =. 71).

**Table 2:** Logistic regression analysis of independent variables affecting the presence of silent brain infarction in primary aldosteronism patients.

Variables			
Dependent	Independent	Adjusted OR (95% CI)	*P* value
SBI	Age	1.15 (1.01–1.38)	0.031*
	SBP	1.03 (0.98–1.09)	0.194
	eGFR	1.00 (0.93–1.07)	0.941
	PRA	0.39 (0.02–1.65)	0.334
	PAC	1.08 (0.99–1.21)	0.100
	K	0.31 (0.03–2.40)	0.262
	HDL-C	0.94 (0.86–1.01)	0.110
	U-aldosterone	0.92 (0.78–1.04)	0.214

SBP = systolic blood pressure, U = urinary

PRA = plasma renin activity, SBI = silent brain infarction,

HDL-C = high-density lipoprotein cholesterol, PAC = plasma aldosterone concentration,

eGFR = estimated glomerular filtration rate,

OR: odds ratio, CI: confidence interval.

**Table 3:** Logistic regression analysis of independent variables affecting the presence of silent brain infarction in primary aldosteronism patients.

Variables			
Dependent	Independent	Adjusted OR (95% CI)	*P* value
SBI	Tissue BOT	0.73 (0.45–0.99)	0.043*
	SBP	1.04 (0.99–1.11)	0.131
	eGFR	0.99 (0.93–1.06)	0.872
	PRA	0.16 (0.00–1.34)	0.187
	PAC	1.10 (1.00–1.23)	0.063
	K	0.47 (0.05–4.26)	0.498
	HDL-C	0.95 (0.87–1.03)	0.215
	U-aldosterone	0.97 (0.81–1.12)	0.665

SBP = systolic blood pressure, U = urinary

PRA = plasma renin activity, SBI = silent brain infarction,

HDL-C = high-density lipoprotein cholesterol, PAC = plasma aldosterone concentration,

BOT = blowout time, eGFR = estimated glomerular filtration rate,

OR: odds ratio, CI: confidence interval.

The association of SBI with age and LSFG findings is shown in Table 4. This analysis divided the patients into four groups based on age (≥ or < 61 years) and tissue BOT (≤ or > 42.0). The occurrence of SBI was significantly different in these groups (*P* <. 001). Among groups including more than 10 patients, the highest occurrence of SBI was 40%, in patients ≥ 61 years old and with tissue BOT ≤ 42.0. SBI occurrence was 29% in patients ≥ 61 years old and with tissue BOT > 42.0. The lowest rate of occurrence was 6%, in patients < 61 years old and with tissue BOT > 42.0.

**Table 4:** Association of silent brain infarction with age and laser speckle flowgraphy findings.

Age (years)	Tissue BOT	Number of patients	Percentage of SBI	*P* value
≧61	≦42.0	10	40.0% (4/10)	＜.001**
	＞42.0	24	29.2% (7/24)	
＜61	≦42.0	1	100.0% (1/1)	
	＞42.0	52	5.8% (3/52)	

Chi-square test

BOT = blowout time, SBI = silent brain infarction

**Fig 2:**
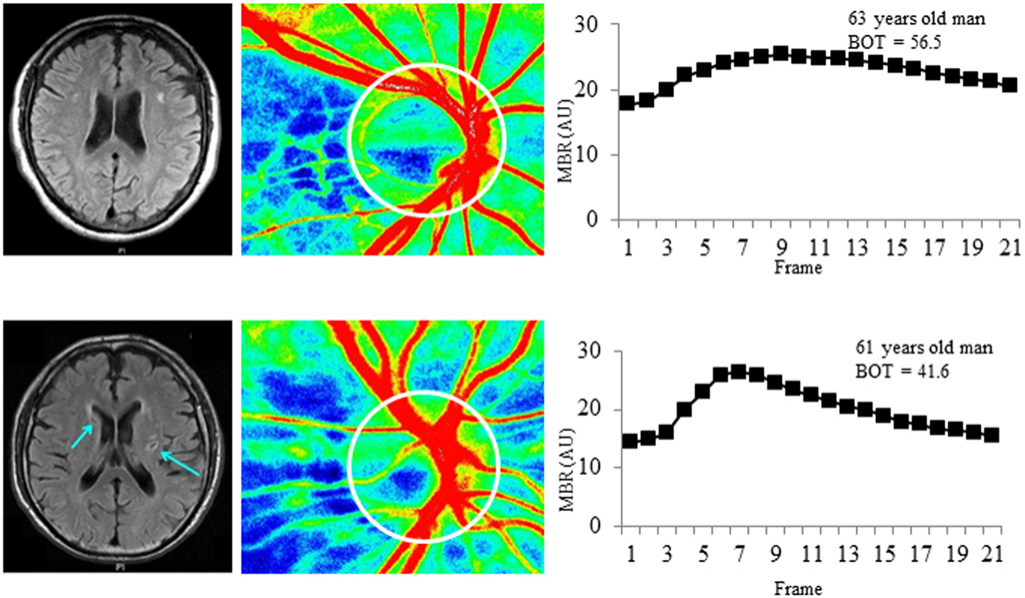
Representative cases with and without silent brain infarction. Above: 63-year-old man without silent brain infarction (SBI). Below: 61-year-old man with SBI. Left: magnetic resonance images; middle: laser speckle flowgraphy (LSFG) color maps; right: LSFG-measured mean blur rate (MBR) waveform. The arrows in the MRI images for the SBI case show regions of clearly visible SBI. The ellipse indicates the optic nerve head (ONH) margin. The LSFG software automatically divided the overall ONH into large vessel and capillary areas. The MBR waveform for the SBI case show that the peak of the waveform is steeper and has a more clearly defined peak than the non-SBI case, which may reflect the effect of age-related vascular changes on the MBR waveform. Furthermore, the values for BOT are 56.5 and 41.6 arbitrary units in the SBI and non-SBI cases, respectively.

Representative PA cases with and without SBI are shown in Fig. 2.

## Discussion

We set out to investigate the relationship between LSFG findings and the occurrence of SBI in patients with PA. We found that the patients with SBI were significantly older and had significantly lower BOT in the capillary area of the ONH than the patients without SBI. Multiple logistic regression analysis revealed that age and BOT were independent factors for the presence of SBI in PA patients. Our analysis showed that Age, with an odds ratio > 1.0, could be regarded as a risk factor for SBI, and tissue BOT, with an odds ratio < 1.0, could be regarded as a protective factor against SBI in patients with PA. Diagnostic criteria combining age and BOT may thus have an excellent ability to predict SBI.

Our study tends to support past findings that age was a risk factor for vascular events in hypertensive patients, including those with PA and EH.[10] As in the earlier report, SBP was higher and male patients were more common in the SBI group included in this study, but we did not find that these were risk factors for SBI. Interestingly, our study also confirmed earlier findings that HDL-C was significantly lower in hypertensive PA and EH patients with a history of cardio- and cerebrovascular events,[10] although again, multiple logistic regression analysis did not show that HDL-C was a protective factor for SBI. A cross-sectional study that included more than 300 healthy subjects also concluded that total serum cholesterol levels were significantly associated with SBI.[31] Nevertheless, we could not find a significant difference in total cholesterol levels between the SBI and non-SBI patients. Furthermore, although we had hypothesized that PAC would be a risk factor for SBI in PA patients,[5,10] and, indeed, we found that PAC tended to be higher in patients with SBI, multiple logistic analysis showed that PAC was not a risk factor for SBI, although only by a narrow margin (*P* = 0.06 in an analysis including tissue BOT, and *P* = 0.10 in an analysis including age). These results differ from those of previous studies, likely because of differences in study aims and subject characteristics. Specifically, our paper investigated risk factors only for SBI, not for symptomatic cerebral infarctions, and only examined patients with PA, not those with with PA and EH.

A previous study reported that a history of stroke, non-fatal myocardial infarction and atrial fibrillation was found in 12.9%, 4.0% and 7.3%, respectively, of patients with PA.[8] Although such cardio- and cerebrovascular events have been reported to be more likely in patients with PA,[8,10] the percentage of PA patients with SBI remained unclear until this study, in which we found that about 20% of PA patients had SBI. The early detection of SBI is still difficult, but important to prevent further cerebral damage, as SBI places a patient at increased risk of both transient ischemic attacks and major strokes.[11] Earlier reports demonstrated the close relationship between retinal microvascular abnormalities and cerebrovascular disorders, and showed that evaluation of the retinal vasculature with tools such as retinal photography is a promising way of measuring the risk of cerebrovascular events.[32,33,34] However, these previously reported methods of retinal evaluation were subjective and lacked clear numeric parameters, creating the possibility of disagreement between different investigators. Thus, it would be desirable to establish new, objective and quantitative biomarkers of SBI using non-invasive methods of evaluating ocular microcirculation.

Ischemic changes after medical interventions are ordinarily evaluated with fluorescein angiography (FA), but this technique is invasive, can cause severe complications, including anaphylactic shock,[35,36] and its results can be affected by time-dependent changes after injection.[37] Recent innovations in LSFG have allowed us to quickly and easily monitor changes in tissue circulation over time.[38,39] The main measurement parameter of LSFG, MBR, is a highly reproducible index of ocular blood flow that correlates well with absolute blood flow values measured with the hydrogen gas clearance and microsphere methods.[40,41,42] Therefore, although MBR was originally a relative measure, in the ONH tissue it can be used for inter-individual and inter-group comparisons.[13] Such comparisons of LSFG measurements have led to many recent findings in glaucoma research, and are especially useful in examining the relationship between glaucoma and ocular circulation.[38,43,44,45,46]

BOT, which we have found to be associated with SBI, is the full duration at half maximum of the MBR waveform, and represents half the duration of a beat. Higher values for BOT indicate that blood flow has a higher volume for a longer time between heartbeats, meaning that blood is supplied well to the peripheral area.[16] The other two parameters, BOS and skew, were not associated with SBI in the current study. BOS functions as an indicator of the volume of blood flow maintained in the vessel between heartbeats, with a higher value indicating that mean blood flow is stably maintained. Skew quantifies the asymmetry of the waveform’s distribution, with a higher value indicating that the distribution of the waveform is shifted leftward. One of the most interesting findings in our study was that although tissue BOT was correlated to age in the non-SBI group, it was not correlated in the SBI group. The reason for this remains unclear, but in general BOT is negatively correlated to age in normal subjects and in patients with atherosclerotic changes.[14,15,16] Although this study evaluated LSFG parameters only from the ONH tissue area, in theory, parameters from the overall ONH, including the large vessels, could also be valuable because of the simplicity of their calculation and the convenience of their use, as had been described earlier.[14,15] However, the fact that BOT in the tissue ONH was associated with SBI supports the conclusion that SBI occurs in the capillaries rather than the large vessels. We therefore believe that SBI may be more likely in older patients in whom PA has caused vascular changes that are relatively stronger than those caused by age. Thus, LSFG measurements of BOT can be considered parameters of vascular age that may prove helpful in evaluating physiological and pathological changes in ocular microcirculation, and become a biomarker of cerebral microcirculation impairment.

Our study was limited by a small sample size (about 90), the lack of patients with EH, and the lack of matching between the groups in age and HDL-C levels. We considered age and tissue BOT separately in our multiple regression analysis, because both the present and previous reports have found these two parameters to be closely correlated. Nevertheless, we believe our results show that in patients with PA, LSFG is currently one of the best choices for identifying the presence of SBI, in addition to age. Furthermore, the combination of LSFG findings with age is a practical way of educating PA patients on their risk of SBI, particularly patients ≥ 61 years of age with tissue BOT ≤ 42.0, as 40% of such patients are likely to have SBI. In addition to age and tissue BOT, our findings suggest that PAC may have potential as a biomarker of SBI, warranting a larger-scale investigation of PA patients.

In conclusion, we found a strong relationship between LSFG findings, particularly the LSFG measurement parameter BOT, and the occurrence of SBI in patients with PA. This suggests that the BOT is potentially a non-invasive, objective biomarker and predictor of SBI, and may be a useful addition to PA evaluations in the future. In particular, since LSFG is a much quicker and simpler measurement technique than MRI, it promises to identify PA patients most at risk of SBI before a definitive determination is made with MRI. Future investigations might usefully build on the findings reported here by examining the role of ocular blood flow in the mechanisms underlying the induction of SBI in PA.
